# Genome-Wide Association Study Confirms Previous Findings of Major Loci Affecting Resistance to *Piscine myocarditis virus* in Atlantic Salmon (*Salmo salar* L.)

**DOI:** 10.3390/genes11060608

**Published:** 2020-05-30

**Authors:** Borghild Hillestad, Ólafur H. Kristjánsson, Shokouh Makvandi-Nejad, Hooman K. Moghadam

**Affiliations:** 1 Benchmark Genetics Norway AS, Sandviksboder 3A, N-5035 Bergen, Norway; borghild.hillestad@bmkgenetics.com; 2 StofnFiskur HF, Staðarberg 2-4, 221 Hafnarfjörður, Iceland; olafur.kristjansson@bmkgenetics.com; 3 Norwegian Veterinary Institute, P.O. Box 750 Sentrum, N-0106 Oslo, Norway; shokouh.makvandi-nejad@vetinst.no

**Keywords:** Atlantic salmon, cardiomyopathy syndrome (CMS), *Piscine myocarditis virus* (PMCV), genome-wide association study (GWAS), single-nucleotide polymorphism (SNP), heritability, breeding

## Abstract

Cardiomyopathy syndrome is a viral disease of Atlantic salmon, mostly affecting fish during the late stages of production, resulting in significant losses to the industry. It has been shown that resistance to this disease has a strong genetic component, with quantitative trait loci (QTL) on chromosomes 27 (Ssa27) and Ssa12 to explain most of the additive genetic variance. Here, by analysing animals from a different year-class and a different population, we further aimed to confirm and narrow down the locations of these QTL. The data support the existence of the two QTL and suggest that the causative mutation on Ssa27 is most likely within the 10–10.5 Mbp segment of this chromosome. This region contains a cluster of major histocompatibility complex class I (MHC I) genes with the most strongly associated marker mapped to one of these loci. On Ssa12, the data confirmed the previous finding that the location of the causative mutation is within the 61.3 to 61.7 Mbp region. This segment contains several immune-related genes, but of particular interest are genes related to MHC II. Together, these findings highlight the likely key role of MHC genes in Atlantic salmon following infection with *Piscine myocarditis virus* (PMCV) and their potential impact on influencing the trajectory of this disease.

## 1. Introduction

Cardiomyopathy syndrome (CMS) is a severe inflammatory cardiac disease of Atlantic salmon (*Salmo salar* L.), mainly affecting farmed animals [1,2,3]. The causative agent, the *Piscine myocarditis virus* (PMCV), is an RNA virus with high similarity to members of the *Totiviridae* family [4,5]. Since the disease was first discovered in 1985, the number of related outbreaks has been on the rise [6,7]. This is a concern for salmon farmers and the associated industry, as it can result in substantial economic losses, considering that the disease mainly affects fish at the late-life stages in seawater [3,8]. In 2003, Brun et al. [1] estimated the annual direct financial loss due to CMS to be between €4.5 and 8.8 million for the Norwegian aquaculture sector. Over the next four years, estimates of these figures increased four- to five-fold [9]. Currently, CMS ranks as one of the top health-related threats to the salmon aquaculture industry [7], and it is considered a disease with severe impacts on animal welfare.

To address these concerns, during the past decade, there have been efforts to understand the host response to PMCV infection. In particular, Timmerhaus et al. [10] provided the first evidence regarding possible genetic differences in resistance to CMS, based on the observed inter-individual variations in the histopathological scores of the affected tissues and the expression profiles of some immune-related candidate genes. In a time-series experiment, they demonstrated that while some fish are characterized by sustained and high loads of the virus along with lesions to the heart, other fish can clear the virus six to ten weeks following infection and carry little pathological evidence of the disease [10]. By profiling and comparing the transcriptomes of these two groups, Timmerhaus et al. [10] further reported an elevation in the expression of genes involved in adaptive immunity among the less-resistant animals. These genes mainly function in T and B cell responses, major histocompatibility complex (MHC) antigen presentation, and apoptosis [10,11]. These data provided the initial indications of a strong genetic basis for resistance against CMS, highlighting the potential role of breeding in helping to mitigate, control, and confine the outbreaks due to this virus.

In this respect, two recent studies have investigated the genetic components and the genomic landscape of PMCV resistance in Atlantic salmon [12,13]. Based on data collected from different populations, the SalmoBreed (https://salmobreed.no) and the Mowi (https://mowi.com/contact/mowi-asa) strains, and different phenotypes, both studies found that the additive genetic variance accounts for a substantial proportion of the phenotypic variation in the host’s response to this disease. The estimated heritability ranged from 0.12–0.38 for mortality data collected from field outbreaks [13] to 0.46, an estimate based on the histopathological scores of the atrium in the Mowi fish infected with the virus in a controlled setting [13], to 0.51, an estimate that was based on loads of the virus in the SalmoBreed strain measured from the apex of the heart, through a quantitative real-time PCR assay and in a challenge infection experiment [12]. Both studies also identified and reported loci that are significantly associated with higher resistance to infection with PMCV [12,13]. In both populations, the genetic markers on chromosome 27 (Ssa27) showed strong association to the resistance status of the animal, irrespective of how the phenotype was measured, whether it was through mortality, histopathology or the viral loads. The two studies have also reported suggestive quantitative trait loci (QTL) on approximately the same region of Ssa12. Collectively, the QTL on these chromosomal segments explained a large proportion of the additive genetic variation in the two investigated populations.

In the current work, we aimed to confirm the presence of the QTL in a different population of Atlantic salmon, the StofnFiskur strain, an Icelandic population with Norwegian origins (http://stofnfiskur.is) [14]. We also investigated the genomics of CMS resistance in a different year-class of the SalmoBreed population. Finally, through a meta-analysis and comparative genomics, we aimed to narrow down the likely positions of the causative mutations on these chromosomal regions and identify candidate genes with likely roles in making animals more resistant against this virus.

## 2. Materials and Methods

### 2.1. Ethics Approval

The challenge trial was approved by the Food and Safety Authority in Norway, Department for National Assignments (FOTS ID: 16666—ref: 18/2433834, approved on 12 December 2018 and FOTS ID: 13515—ref: 17/180215, approved on 23 October 2017), which conforms to the Guide for the Care and Use of Laboratory Animals published by the US National Institutes of Health (NIH Publication 85-23, revised 1996).

### 2.2. Infection Challenge Tests in the SalmoBreed and StofnFiskur Populations

We previously outlined the details for the CMS infection challenge procedure for the SalmoBreed 2017 year-class (SB-17), along with the sample collection for genotyping and viral load assessment [12]. In that study, we challenged about 1100 passive integrated transponder tagged (PIT-tagged) smolts from 60 full-sib families. In the current investigation, our approach was the same as before [12], where we infected 1235 SalmoBreed smolts from the 2018 year-class (SB-18), representing 153 families, as well as 1849 fish from the StofnFiskur 2017 year-class (SF-17), cohort two, representing 150 families. The experiments were conducted at the VESO Vikan challenge facility (https://www.veso.no/about-us; Namsos, Norway). In brief, following extraction and homogenization of spleen, head kidney and heart from PMCV-infected fish (mean cycle threshold (*Ct*) value of 24.5), the homogenate was stored in L15 media, containing 50 µg/mL gentamicin (final dilution of 1:10). The homogenate was further tested for the absence of other pathogens such as *Piscine orthoreovirus*, *Infectious pancreatic necrosis virus* and *Salmonid alphavirus*. The fish were infected through intraperitoneal injection (0.1 mL per fish) and then transferred to a single tank where animals also functioned as shedders. The trials were carried out for nine weeks. By the termination of the trial, there was no mortality due to infection. The heart apex was collected and stored in RNALater. PatoGen AS (http://www.patogen.com/; Ålesund Norway) conducted the RNA extraction, target sequence amplification and calculations of normalised *Ct* values. However, unfortunately, due to issues related to competing patents, it could not reveal any details of these procedures.

### 2.3. Genotyping, Estimation of Genetic Parameters and Genome-Wide Association Study

Following the collection of the adipose fin-clips, samples were sent to IdentiGEN (https://identigen.com/; Dublin, Ireland) for DNA extraction and genotyping. Genotyping was done on a custom-made, 55k Affymetrix Axiom array. The initial quality control and single-nucleotide polymorphism (SNP) calling steps were done with the Affymetrix Axiom analysis suite software. Additional filtering was implemented using PLINK version 1.9 [15], where we removed the low-quality genotyped samples and SNPs by setting call rates < 95%, Hardy–Weinberg *p*-value < 10^−10^ and minor allele frequency < 0.05%. The estimations of variance components and SNP-based heritability, as well as the genome-wide association study (GWAS), were performed using restricted maximum likelihood and a mixed linear model, implemented in the Genome-wide Complex Trait Analysis (GCTA) software v1.93 [16]. The model used for estimating variance components was as follows:

*y* = X*b* + Z*c* + Z*g* + *e*, (1)

where *y* is the vector of *Ct* values of the viral load, X and Z are the incidence matrices assigning phenotype to the vectors of fixed effects (*b*), that is, the number of days from hatch to termination, weight at termination, the first five eigenvectors from the principal component analysis and the gender effect, predicted based on the presence or absence of Y-specific genetic markers [17], the common-environmental family effect (*c*), the animal genomic breeding value (*g*) and the residuals (*e*). The effects common to families, animal and residuals are assumed to follow a normal distribution, that is, $c\;\sim \;N\left(0,\;I\sigma_{c}^{2}\right)$, $u\;\sim \;N\left(0,\;G\sigma_{g}^{2}\right)$ and $e\;\sim \;N\left(0,\;I\sigma_{e}^{2}\right)$*,* where I is an identity matrix, G is the genomic relationship matrix, computed according to Yang et al. [16], and $\sigma_{c}^{2}$, $\sigma_{g}^{2}$ and $\sigma_{e}^{2}$ are the variances of full-sib family effect, additive genetic and residuals respectively.

The genome-wide association was performed using a model similar to the one described above, except we also fitted the allele substitution effect for each SNP. The model used for the analysis can generally be described as:

*y* = X*b* + W*a* + Z*g* + *e*, (2)

where *y*, X, *b*, Z, *g* and *e* are the same as above, W is the incidence matrix for SNP genotypes and *a* is the allele substitution effect. For the pooled analysis, the year-class was also included as a fixed effect. An association was considered to be genome-wide or chromosome-wide significant if the *p*-values were less than the adjusted significance threshold of 0.05/*NG* or 0.05/*NC,* where *NG* and *NC* are the number of genetic markers across the genome or the total number of SNPs per chromosome, respectively [18,19]. The genomic inflation factor (*λ*) was calculated using the equation described by Utsunomiya et al. [20] as $median(\left(a^{2}/se^{2}{)}^{2}\right)/0.456$, where *a* is the allele substitution effect of an SNP and *se* is its respective standard error. The proportions of the phenotypic (*V_P_*) and the genetic (*V_g_*) variances explained by each marker were computed from the estimated *a* and the frequency of the alleles (*p*) as $V_{P}=2p_{i}\left(1-p_{i}\right)a_{i}^{2}/\sigma_{P}^{2}$ and $V_{g}=2p_{i}\left(1-p_{i}\right)a_{i}^{2}/\sigma_{g}^{2}$.

## 3. Results and Discussion

**Figure 1:**
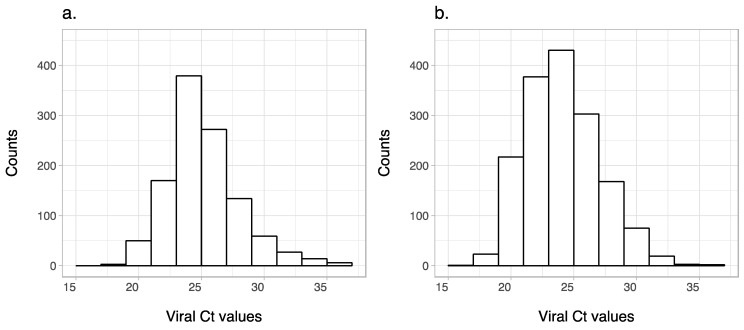
Cycle threshold (*Ct*) values of *Piscine myocarditis virus* (PMCV) loads detected from the heart of infected fish from (**a**) SalmoBreed and (**b**) StofnFiskur populations. Note that *Ct* values are inversely related to the level of the virus detected from the tissue.

We observed considerable inter-individual variation in the level of the virus found from the apex of the heart, with a median *Ct* value of 25, minimum of 15 and a cut-off of 37 (Figure 1). Previously, we and others [5,11,12] have shown that the cardiac *Ct* values of PMCV-infected fish are highly correlated with the histopathological scores for both atrium and ventricle (Spearman *r* = 0.76, *p*-value < 0.0001). Such a high correlation suggests that viral load is an accurate proxy for assessing the degree of damage to the heart, and hence the resistance potential of an animal to this disease. Two independent studies provided further support, indicating that these different ways of phenotypic recording (i.e., *Ct* and histopathology scores) for this trait most likely point to the same underlying genetic mechanism of resistance. This can be concluded as both studies, one through measuring the viral load [12] and the other by assessing the histopathological damages to the heart as well as scoring the dead and alive fish from a field outbreak [13], found significant associations on the same regions of the genome.

**Figure 2:**
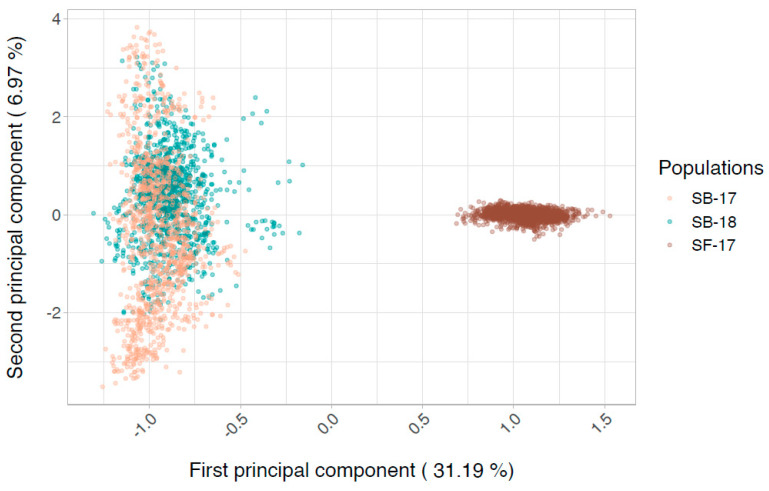
Principal component analysis of the genomic data from different populations of Atlantic salmon, SalmoBreed and StofnFiskur.

In the current study, following the exclusion of animals and SNPs that did not pass the quality control threshold filtering, there were 1114 fish from the SB-18 population, 1618 from the SF-17 population and approximately 49.5k SNPs for the genomics assessment. Further, for the meta-analysis, an additional 1099 fish from the SB-17 year-class were included. The principal component analysis (PCA) of the genomic data confirmed that the StofnFiskur and the SalmoBreed fish investigated in this study constitute distinct clusters, as they were separated based on the origins of the fish (Figure 2). The SNP-based *h^2^* was estimated to be 0.57 ± 0.12 for the SB-18 population and 0.39 ± 0.07 for the SF-17 population (Table 1). These estimates are comparable to our previous estimate of 0.51 (SB-17) [12] and to that reported by Boison et al. [13], 0.46.

**Figure 3:**
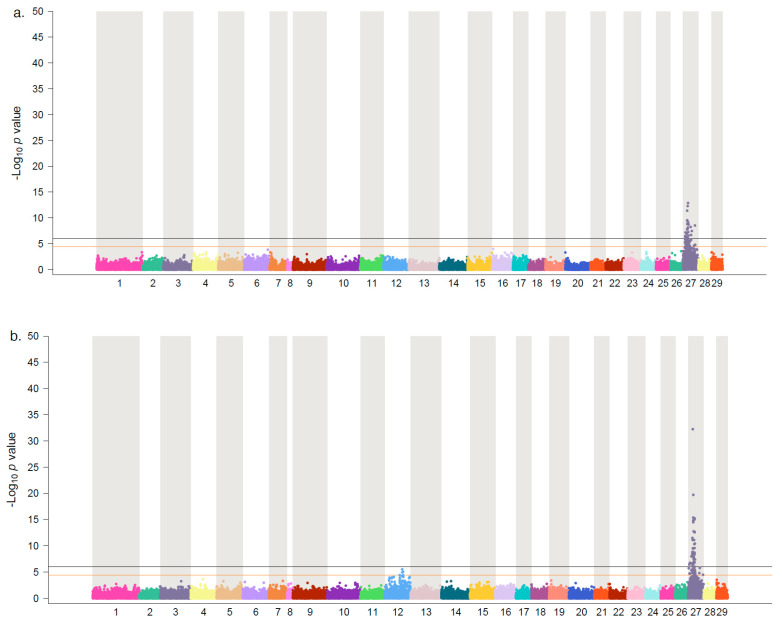

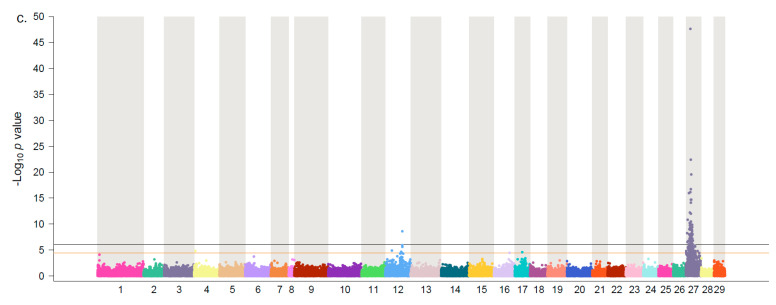
Manhattan plot of association analysis to CMS resistance in Atlantic salmon. The figures show the −*log_10_ p*-value of the test statistics for each SNP plotted against the physical positions of the markers on the chromosomes. The black and orange lines indicate the genome-wide and chromosome-wide significance threshold cut-off levels, respectively. (**a**) SalmoBreed population, year-class 2018 (SB-18); (**b**) StofnFiskur 2017 year-class (SF-17), and (**c**) meta-analysis based on three populations, SB-17, SB-18 and SF-17.

In both SB-18 (*λ* = 0.9) and SF-17 (*λ*= 1.02), we identified significant associations between markers on Ssa27 with the levels of the virus detected from the heart (Figure 3a,b). Previously it was shown that the causative mutation on Ssa27 is most likely located in the 8.5–12 Mpb region of this chromosome [12,13]. In this work, the SNP with the lowest associated *p*-value in both populations was located at Ssa27: 10,052,153 bp. In SB-18, this SNP explained about 8% of the phenotypic variations and 13.5% of the genetic variations (Table 2). In SF-17, the same marker explained approximately 16% of the phenotypic variations and more than 38% of the genetic variations (Table 2). The SNP is in the 3’ untranslated region (UTR) of a major histocompatibility complex (MHC) class I-related gene (LOC106588402) (Supplementary Table S1). The second most strongly linked QTL markers were located at 10.34 (Ssa27: 10,348,407 bp) and 10.41 (Ssa27: 10,413,738 bp) Mbp in SB-18 and SF-18 respectively. The marker on 10.34 Mbp was also reported as one of the most significantly associated markers to CMS QTL in the SB-17 [12]. The SNP is in the 3’ UTR of a solute-carrier 39 like gene (LOC106588393). The SNP on 10.41 Mbp is in the coding sequence of an uncharacterised gene (LOC106588383). The variation is a missense-substitution that converts asparagine to aspartic acid. The predicted protein of this gene contains a domain similar to immunoglobulin V-set (protein family: PF07686), suggesting that this gene product is most likely involved in the immune system machinery of the fish.

The meta-analysis consisted of 3804 animals, allowing us to increase the power of QTL detection. As expected, we found a strong association on Ssa27, with 65 markers passing the genome-wide Bonferroni threshold on this chromosome (*λ*= 1.07; Figure 3c). The most strongly linked marker was the same as the top SNP identified in SB-18 and SF17, located at Ssa27: 10,052,153 bp, with a *−log_10_ p*-value of 48. The SNP with the second-lowest *p*-value mapped to Ssa27: 10,348,407 bp with a *−log_10_ p*-value of 22.5. Collectively, data presented in this study, based on individual populations as well as the pool of all animals, along with our previous finding [12] point to the 10–10.5 Mbp region of Ssa27 as the most likely segment on this chromosome containing the causative mutation(s). This region of Ssa27 harbours a cluster of MHC class I genes (Supplementary Table S1). Different studies have highlighted the importance of MHC gene products in increasing the resistance of salmon against several pathogenic agents [21,22]. In this regard, Timmerhaus et al. [10,11] previously identified a set of transcripts involved in the presentation of viral antigens through MHC class I and II genes, with different expression profiles between the naïve and PMCV-infected fish, as well as among the infected animals. We have reported that the genes associated with a few of these transcripts map to this QTL genomic segment [12]. Therefore, it is likely that the causative variation(s) are associated with the MHC-related gene(s), causing changes in the levels of expression or modifications in the structural properties of its protein.

Previously, the presence of a suggestive QTL on Ssa12 was also reported [12,13]. The position of QTL was estimated to fall within the 61–63 Mbp region of this chromosome. In particular, in SB-17, we showed the strongest association to markers within a 320 kbp segment, from 61.39 to 61.71 Mbp. In this region, there are two putative H-2 class II histocompatibility antigen genes and a putative transcription factor EB (Supplementary Table S1). The product of the latter gene mainly functions in the elimination of intracellular pathogens, the reduction of inflammation, antigen presentation and the secretion of cytokines [23]. In the current study, we did not find evidence of QTL on Ssa12 in SB-18, suggesting that the QTL in this population is most likely not segregating (Figure 3a). In SF-17, on the other hand, three markers passed the chromosome-wide Bonferroni threshold level, with the two top markers mapped to Ssa12: 61,626,398 bp and Ssa12: 61,694,062 bp (Figure 3b). The first SNP is an intronic variant located in the transcription factor EB-like gene (LOC106565694), explaining 2.71% and 6.70% of the phenotypic and genetic variations respectively. The second SNP is in the 3’ UTR of the H-2 class II histocompatibility antigen, I-E beta chain gene (LOC100136577). In the pooled analysis of all populations, however, this SNP, with a *−log_10_ p*-value of 8.60 was most strongly associated with the QTL (Figure 3c). This was followed by an intergenic variation at Ssa12: 61,443,087 bp (*−log_10_ p*-value of 5.92) and an SNP at the 3′ UTR of H-2 class II histocompatibility antigen, A-U alpha chain-like gene (LOC106565699) at position Ssa12: 61,702,164 bp (*−log_10_ p*-value of 5.59). These findings further support our previous estimate of the approximate location of the QTL on this chromosome.

In conclusion, this work confirms the presence of at least two different genomic loci with a substantial effect on the resistance of Atlantic salmon to PMCV infection. We narrowed down the locations of these QTL and suggested candidate genes within these regions. In particular, our findings imply that the MHC classes I and II are likely to play crucial roles in the host immune system following infection with this virus, and can be an essential determinant of the outcome of the disease.

## Figures and Tables

**Table 1:** Variance components and heritability estimates for cardiomyopathy syndrome (CMS) resistance in the two Atlantic salmon populations investigated in this study, StofnFiskur year-class 2017 (SF-17) and SalmoBreed year-class 2018 (SB-18).

Parameters	SF-17	SB-18
$\sigma_{g}^{2}$ (*se*)	3.11 (0.63)	3.62 (0.80)
$\sigma_{c}^{2}$ (*se*)	1.50 (1.13)	4.28 (1.75)
$\sigma_{e}^{2}$ (*se*)	3.29 (0.54)	6.98 (0.98)
$\sigma_{P}^{2}$ (*se*)	7.91 (0.65)	6.31 (0.94)
$h^{2}$ (*se*)	0.39 (0.07)	0.57 (0.12)

$\sigma_{g}^{2}$: genetic variance; $\sigma_{c}^{2}$: common environmental variance; $\sigma_{e}^{2}$: residual variance; $\sigma_{p}^{2}$: phenotypic variance; $h^{2}$: heritability; *se*: standard error.

**Table 2:** Summary statistics of the top two quantitative trait loci (QTL) associated single-nucleotide polymorphisms (SNPs) per-population that passed the genome-wide or chromosome-wide significance threshold.

Population	chr	Position	*p*-Value	maf	*V_P_*	*V_g_*
SB-17	27	10,393,267	1.63 × 10^−14^	0.32	7.69	15.62
SB-17	27	10,348,407	4.51 × 10^−15^	0.48	7.71	15.68
SB-18	27	10,052,153	2.18 × 10^−13^	0.17	7.97	13.55
SB-18	27	10,348,407	5.64 × 10^−13^	0.50	8.30	14.11
SF-17	27	10,052,153	1.51 × 10^−35^	0.39	15.70	38.80
SF-17	27	10,413,738	3.65 × 10^−21^	0.37	9.17	22.65
Meta-analysis	27	10,052,153	2.06 × 10^−48^	0.27	9.57	21.91
Meta-analysis	27	10,348,407	3.91 × 10^−23^	0.49	4.49	10.29
SB-17	12	61,702,164	9.65 × 10^−07^	0.12	3.03	6.16
SB-17	12	61,701,806	5.74 × 10^−06^	0.12	2.64	5.36
SF-17	12	61,626,398	1.38 × 10^−06^	0.22	2.71	6.70
SF-17	12	61,694,062	2.97 × 10^−06^	0.39	2.87	7.09
Meta-analysis	12	61,626,398	2.47 × 10^−09^	0.41	2.34	5.36
Meta-analysis	12	61,443,087	1.20 × 10^−06^	0.20	1.17	2.68

chr: chromosome; maf: minor allele frequency; *V_P_*: phenotypic and *V_g_*: genetic variances explained by the mark.

## Supplementary Materials

The following are available online at https://www.mdpi.com/2073-4425/11/6/608/s1, Table S1: List of genes within the two QTL regions reported in this study along with their putative products and functions.
